# Modeled Tracheidograms Disclose Drought Influence on *Pinus sylvestris* Tree-Rings Structure From Siberian Forest-Steppe

**DOI:** 10.3389/fpls.2018.01144

**Published:** 2018-08-06

**Authors:** Margarita I. Popkova, Eugene A. Vaganov, Vladimir V. Shishov, Elena A. Babushkina, Sergio Rossi, Marina V. Fonti, Patrick Fonti

**Affiliations:** ^1^Department of Mathematical Methods and Information Technology, Siberian Federal University, Krasnoyarsk, Russia; ^2^Siberian Federal University, Rectorate, Krasnoyarsk, Russia; ^3^V.N. Sukachev Institute of Forest SB RAS, Krasnoyarsk, Russia; ^4^LE STUDIUM Loire Valley Institute for Advanced Studies, Orléans, France; ^5^Khakassia Technical Institute, Siberian Federal University, Abakan, Russia; ^6^Département des Sciences Fondamentales, Université du Québec à Chicoutimi, Chicoutimi, QC, Canada; ^7^Key Laboratory of Vegetation Restoration and Management of Degraded Ecosystems, Guangdong Provincial Key Laboratory of Applied Botany, South China Botanical Garden, Chinese Academy of Sciences, Guangzhou, China; ^8^Institute of Ecology and Geography, Siberian Federal University, Krasnoyarsk, Russia; ^9^WSL Swiss Federal Research Institute, Landscape Dynamics, Birmensdorf, Switzerland

**Keywords:** cambial activity, cell size, process-based Vaganov-Shashkin model, South Siberia, tracheid, tracheidogram, VS-oscilloscope

## Abstract

Wood formation allows trees to adjust in a changing climate. Understanding what determine its adjustment is crucial to evaluate impacts of climatic changes on trees and forests growth. Despite efforts to characterize wood formation, little is known on its impact on the xylem cellular structure. In this study we apply the Vaganov-Shashkin model to generate synthetic tracheidograms and verify its use to investigate the formation of intra-annual density fluctuations (IADF), one of the most frequent climate tree-ring markers in drought-exposed sites. Results indicate that the model can produce realistic tracheidograms, except for narrow rings (<1 mm), when cambial activity stops due to an excess of drought or a lack of growth vigor. These observations suggest that IADFs are caused by a release of drought limitation to cells formation in the first half of the growing season, but that narrow rings are indicators of an even more extreme and persistent water stress. Taking the example of IADFs formation, this study demonstrated that the Vaganov-Shashkin model is a useful tool to study the climatic impact on tree-ring structures. The ability to produce synthetic tracheidogram represents an unavoidable step to link climate to tree growth and xylem functioning under future scenarios.

## Introduction

As long-living and sessile organisms, wooden plants needs to continuously adjust their structure to survive under changing environmental conditions. These adjustments are usually achieved while growing (Meinzer et al., 2011). Thanks to the primary and secondary meristems, plants are indeed constantly producing new tissues when conditions are favorable (Vaganov et al., 2006). The cambium can modify the number of cells produced, and their morphological characteristics to regulate the important xylem and phloem functions of transport, storage, and mechanical support. In this way, not only the tissues are constantly renewed, but also their form and functioning. This capacity offers trees the necessary dynamic to face changes. However, since wood formation requires resources and time to take place (Steppe et al., 2015), the current environment conditions strongly influence the width, structure and chemical composition of the annual rings, thus limiting the ability of plants to acclimate and endure. These limitations can restrict the physiology over several years, as already observed as a consequence of increasing drought pressure (Anderegg et al., 2013; Britez et al., 2014), threatening plant survival (McDowell et al., 2008) and eventually leading to forest mortality (Allen et al., 2010).

A better understanding of the chain between environment, tree-physiology, wood formation, wood structure and plant performance is unavoidable to soundly assess the fate of trees species and provenances under a rapidly changing environment (Sass-Klaassen et al., 2016). Without a clear long-term and high-resolved perspective of the process of xylem formation and its interaction with the environment, it is not possible to fully comprehend how annual growth rings and their typical wood structures are formed and respond to climate and their extreme events (Pacheco et al., 2016; Rathgeber et al., 2016). Evidences of the impact of the environment on plant growth are not only manifold, but are also exploited to reconstruct past environmental conditions via the study of tree-rings (Fritts, 1976). Novel methods over the last decades have extended interests into the processes of tree-ring formation toward higher intra-annual resolution and deeper mechanistic understanding of environmental impact (McCarroll and Loader, 2004; Fonti et al., 2010). Attention has also been given to the process of xylogenesis (e.g., Cuny et al., 2015) to better assess cambial phenology (e.g., Rossi et al., 2016), timing (Carrer et al., 2017) and dynamic (Cuny et al., 2014) of growth. Based on the improved spatial (cellular) and temporal (weekly) resolution, it has now become possible to better link the impact of specific environmental events to the processes shaping the amount of carbon fixed (Cuny et al., 2015) and its cellular structure (e.g., Abrantes et al., 2013; Castagneri et al., 2015), as well as its influences on the functioning of the xylem (e.g., Mayr et al., 2006; Martin-Benito et al., 2017).

Intra-annual density fluctuations (IADF), i.e., a density anomaly appearing within the annual ring due to the occurrence of latewood-like cells within earlywood, or earlywood-like cells within latewood, represent one of the most obvious examples of climatic impact on the tree-ring structure (Fritts, 1976). IADFs are in general associated to unusual strong events (as drought or cold period) changing the “typical” process of cell development” as e.g., a temporary decrease in growth rate. Its occurrence has been suggested to be associated with plastic adjustments to maintain the balance between hydraulic efficiency and safety under short-term variations in environmental conditions (De Micco et al., 2016). Despite numerous investigations (e.g., Nabais et al., 2014), reliability of IADFs occurrence and distribution is still uncertain (Campelo et al., 2015). IADFs do not arise in all the trees within the same site, not even all drought events trigger IADFs. This variability has been associated to differences in sensitivity among species (Pacheco et al., 2016), tree size and age (Campelo et al., 2015), growth rate (Rigling et al., 2001), sex (Olivar et al., 2015), or to difference in intensity and duration of the climatic event (Vieira et al., 2017).

To better assess the ability of tree species to acclimate and endure a changing environment requires tools allowing projecting growth under future climatic scenarios (Guiot et al., 2014). Process-based models of tree-ring growth [e.g., *MuSICA* (Ogee et al., 2003); *CASSIA* (Schiestl-Aalto et al., 2015); and *CASTANEA* (Delpierre et al., 2012)] provide this additional perspective for simulating intra-annual growth under differing climatic scenario as increasing intensity and duration of drought (e.g., Wilkinson et al., 2015). Several process-based model exists to estimate the annual course of wood biomass, which is essential to study the impact of intra-seasonal climatic event on tree-growth. However, these models are usually not able to provide data on the formed wood structure [with few exception as Deleuze and Houllier (1998) and *CAMBIUM* model by Downes et al. (2009)] – one of the most critical connections between environment and plant functioning – precluding the opportunity to connect structure to function. The Vaganov-Shashkin model (*VS-model*, Vaganov et al., 2006) is an environmental driven conifer tree-ring growth model that has proven to provide reliable estimates under strong limited conditions (for examples of model application see Touchan et al. (2012), Shishov et al. (2016), Yang et al. (2017), and for local use see e.g., Arzac et al., 2018). A singularity of this model is the assumption that the environment (via the most limiting environmental factor) is determining xylem cell differentiation (cell enlargement and wall thickening) only during the time-window the cells are residing in the cambial zone. In this way, the model computes daily growth rate relative to the growth in absence of limitations. To run, the model only requires daily temperature and precipitation data and needs to be calibrated to the growth characteristics for the selected species specific to the site considered (Vaganov et al., 2006; Shishov et al., 2016).

In this study we apply the process-based VS-model to simulate intra-annual cell anatomy, thus providing a novel tool to link environment with wood structure and functioning. In particular, we aim to (i) verify if the VS-model provides realistic timing and growth rates in *Pinus sylvestris* trees growing in a drought sensitive area in southern Siberia; (ii) verify if the VS-model is able to reproduce tracheidograms of radial tracheid diameter including IADF; and (iii) apply VS-model to identify if dry weather conditions in summer produce IADF in *P. sylvestris*.

## Materials and Methods

### Study Site and Climate

This study uses wood cores collected in late 2013 on a foothill-forested site around Malaya Minusa (53°43′ N, 91°47′ E, 251 m a.s.l.), southern Siberia (Russia), a village close to Minusinsk at the border with the Altai–Sayan region. The area is a steppe-like landscape characterized by a cold and semi-arid climate. Records from the 25 km close meteorological station of Minusinsk (53°41′ N, 91°40′ E, 254 m a.s.l.) indicated an average annual temperature of 1.0°C and precipitation sum of 341 mm (average 1936–2013), 90% of which falls from the end of April to the beginning of October. The site is composed of a mixed forest of birch (*Betula pendula*) and pine (*P. sylvestris*) growing on deep sandy soil covered by sedge-grass and mosses.

### Tree-Ring Width and Tracheidograms

The wood cores were collected from a selection of 20 damage-free, dominant and mature *P. sylvestris* trees. Two parallel radial cores were extracted at stem breast height from each tree using an increment borer with a diameter of 5 mm. Half of the cores (one per tree) were prepared to build an annual growth chronology– necessary to calibrate the VS-model – and the second half served the description of the tracheid anatomical properties. Ring widths were measured with a precision of 0.01 mm using a LINTAB measuring table connected to the TSAP software (Rinntech, Heidelberg, Germany). The obtained time-series were visually cross-dated and verified with the software *COFECHA* (Grissino-Mayer, 2001). A 50%-variance cubic smoothing spline with a 2/3 cut-off was used to remove non-climatic factors and the auto-correlations removed with an auto-regressive modeling. The residual tree-ring chronology (1936–2013) was finally obtained by averaging the individual time-series with a bi-weight robust mean (Cook and Kairiukstis, 1990).

Tracheidograms were built to characterize the variation of tracheid radial size along the annual rings (Tychkov et al., unpublished). The cores from the five trees best correlating with the chronology (*R* > 0.70) were selected. Cell measurements were performed on cross-section images of safranin-stained micro-sections (15–20 μm thick) cut with a sliding microtome (Reichert, Germany) along the 50 last annual rings (1964–2013). The images were captured at a 400 × magnification with a digital camera (5 Megapixels) connected to a microscope (Axio Imager A1m, Carl Zeiss, Germany). Tracheids radial cell diameter (TD) were assessed by measuring the lumen radial diameter (LD) and double tangential cell wall thickness (2CWT) along five undisturbed and representative radial files in each ring (Vaganov et al., 2006; Seo et al., 2014) using the image analysis package *Lineyka, SuperMoment*, and *ProcessorKR* (Silkin, 2010). All measured files were standardized, i.e., adjusted to the rounded average cell number for the trees in that year (Vaganov et al., 1985; Campelo et al., 2016).

**FIGURE 1:**
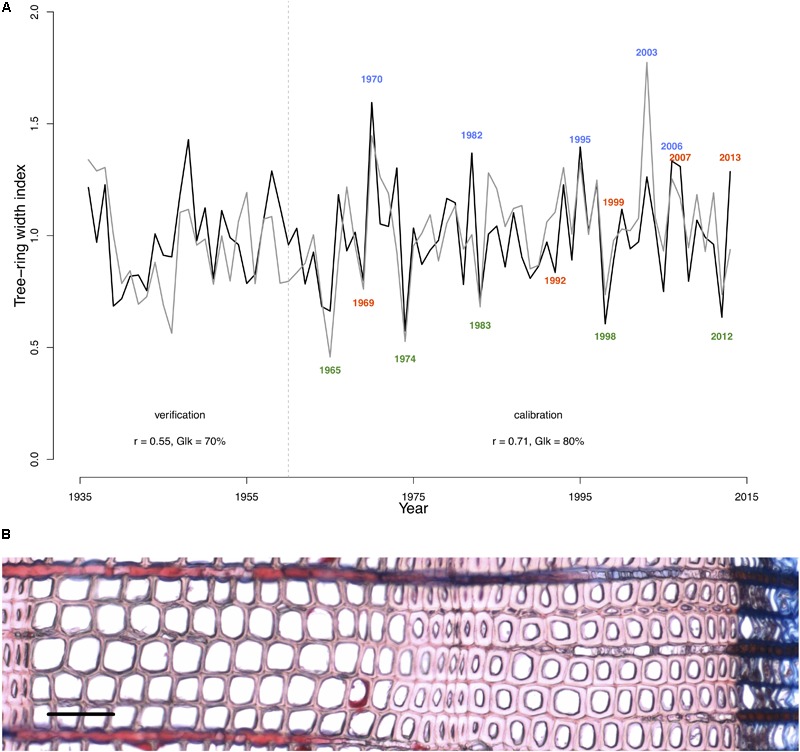
Model calibration and selection of ring types. **(A)** The gray line indicates the detrended Pinus sylvestris tree-ring chronology over the period 1936–2013 at the Malaya Minusa in southern Siberia; the black one shows the growth rate obtained with the model. Pearson’s correlations (*r*) and Gleichläufigkeit (Glk) for both the calibration (1960–2013) and verification period (1936–1959) are indicated. Colored years indicate the annual rings grouping [N = narrow rings (green); W = wide rings (blue); I = IADF rings (red)] according to their typical tree-ring pattern. **(B)** Images of a ring displaying a typical IADF (ring 2013, scale = 100 μm).

**FIGURE 2:**
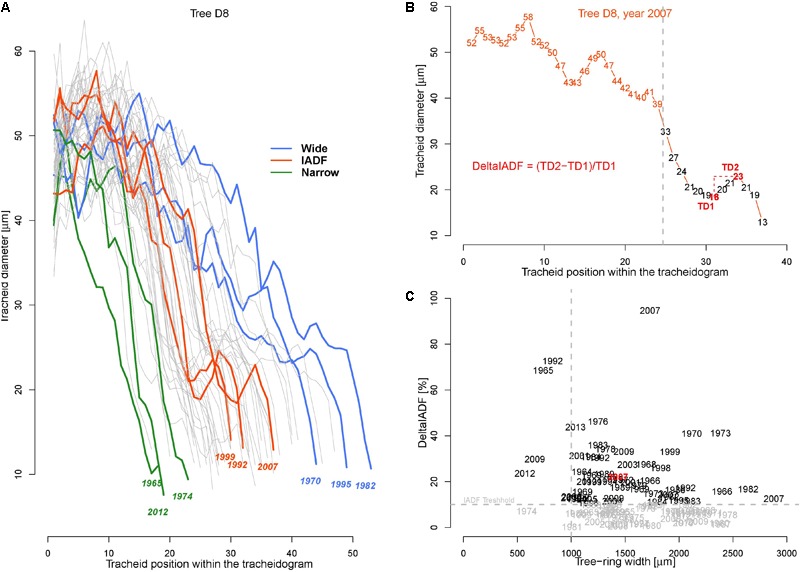
Tracheidograms and intra-annual density fluctuations (IADF). **(A)** Overview of all 50 tracheidograms of tracheid diameter for the annual rings of tree D8 with different ring types shown in colors [N = narrow rings (green); W = wide rings (blue); I = IADF rings (red), see **Figures 1** and **B**]. **(B)** Example of tracheidogram displaying a L+IADF (numbers indicate the tracheid diameter). L+IADFs have been defined in this study as rings showing a tracheid size increase of at least 10% occurring in the last third of the tracheidogram. The formula in red indicates how the increase of tracheid diameter (DeltaIADF) has been assessed. **(C)** Presence of DeltaIADFs as a function of tree-ring width for all the 250 annual rings considered in the study. In black are indicated the years of the annual ring showing a L+IADF (i.e., with DeltaIADF > 10%). The year in red corresponds to the example of **B**.

To perform analysis, from the 50 available calendar years, we selected three groups of years inducing specific tree-ring anatomical patterns (ring types), namely the narrow (N, 1965, 1974, 1983, 1998, 2012), wide (W, 1970, 1982, 1995, 2003, 2006), and IADF (I, 1969, 1992, 1999, 2007, 2013) rings. The selection of narrow and wide ring was based on the 5 years in the chronology with, respectively, the largest and smallest ring width (see **Figure 1**). The IADF have been based on the presence of IADF from the latewood type L+ (Campelo et al., 2007), i.e., density fluctuation occurring in the latewood, the most common at the selected site. These patterns have been identified using the tracheidograms and specifically when tracheid radial size in the second half of the ring showed an increase of >10% compared to a previous local minima (**Figure 2C**).

### Xylogenesis and Tracheid Anatomical Measurements

To monitor seasonal tree-ring growth, microcores from another 15 trees from the same forest were collected at the stem breast height from April to October 2013 (with approx. a 10-days interval for a total of 18 sampling dates). Trees characteristics are shown in **Table 1**. Samples were fixed in water-glycerin-ethanol solution (1:1:1) to maintain the soft tissue in their original hydrated status. Cross-sections (15-μm thick) were obtained with a sliding microtome (Thermo Fisher Scientific HM 450), stained with safranin (1% solution) and astra blue (2% solution) and placed into glycerin on a microscope slide. Growth was assessed by counting along three radial files the tracheids in the cambial, enlargement, wall thickening, and mature zone. The last collected micro-sections were used to characterize tracheid anatomy, by measuring the radial lumen size and the double cell wall thickness along five radial files using AxioVision (SE64 Rel. 4.9.1, Carl Zeiss, Jena, Germany).

**Table 1:** Trees and tree-ring characteristics.

	H (m)	DBH (cm)	Age (years)	TRW (mm)			Ncell			TD (μm)		
	Mean	Mean	Mean	Mean	Min	Max	Mean	Min	Max	Mean	Min	Max
All 20 trees	17.8	37.5	93	1.46	0.59	3.22	–	–	–	–	–	–
Five trees	18.2	35.8	95	1.33	0.22	2.88	38	9	76	35.0	18.7	45.5
Tree1	18	40	90	1.75	1.05	2.88	44	29	71	40.2	36.2	45.5
Tree2	17	26	91	1,69	0.69	2.64	49	23	76	33.9	29.0	37.2
Tree5	20	41	90	0.73	0.22	2.51	21	9	70	33.4	18.7	38.9
Tree7	20	43	111	1.27	0.57	2.38	43	23	73	29.0	23.1	33.7
Tree8	16	29	92	1.22	0.51	1.99	32	17	53	38.4	28.5	44.8
15 trees monitored	15	25	36	2.46	0.16	10.4	–	–	–	–	–	–

*H = Tree height; DBH = Stem diameter at breast height; TRW = tree-ring width; Ncell = number of cells in tracheidogram; TD = Cross-sectional tracheid radial diameter.*

### VS-Model Calibration and Assessment of Daily Growth Rates

The VS-model computes relative daily growth rate (Gr) from daily data of temperature, precipitation and hours of sunlight. These computations require the calibration of the most sensitive parameters to the growing characteristics of the species at the site (Tychkov et al., unpublished). We performed the model calibration (1960–2013) and verification (1936–1959) by comparing the simulated time series of annual growth rates with the site-specific detrended tree-ring width chronology using the VS-oscilloscope (Shishov et al., 2016). This tool is specifically designed to interactively adjust the model parameters and directly visualize and assess the match between the model output and the indexed tree-ring width chronology. As input for the model we used the daily temperature and precipitation data from the meteorological station of Minusinsk (53°41′ N, 91°40′ E, 254 m a.s.l.) from January 1936 to December 2013.

### Linking Growth Rates to Tracheid Radial Diameter

**FIGURE 3:**
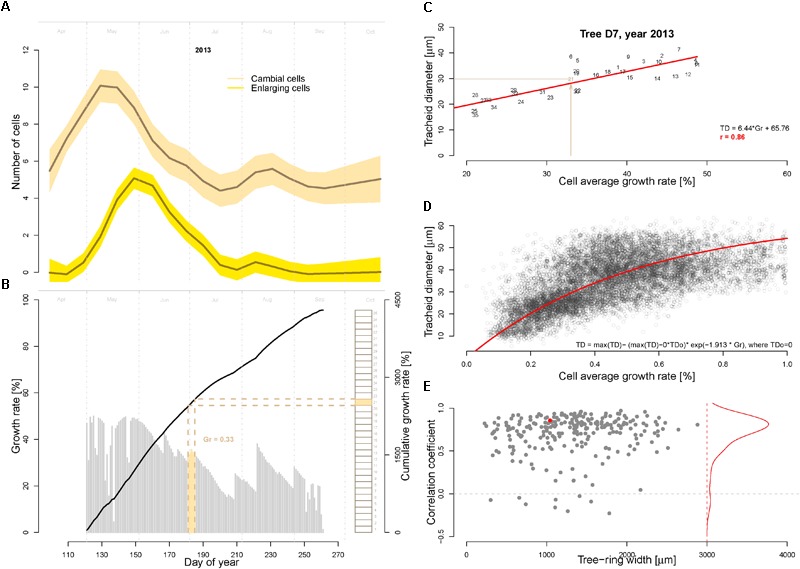
Assessment of cambial cell growth rates and its relationship to the tracheid diameter. **(A)** Average number of cambial (beige) and enlarging cells (yellow) as observed during the growing season 2013 with repeated micro-coring on 15 trees from the study site in Malaya Minusa. **(B)** Daily (gray bars) and cumulated (black thick line) growth rates as assessed by the calibrated model for the growing season 2013. Brown rectangles on the right indicate the sequentially developing cambial cells (*n* = 31 for tree D7, assuming that the production of a successive cambium cell only occurs when the previous one is completed) used to assess the timing of their development and the corresponding average cell growth rate (the filled rectangle correspond to the 21st cambial cell). Specifically, the timing of each dividing cambial cell [i.e., when environmental conditions determine the future cell differentiation, see (Vaganov et al., 2006)] was assigned by dividing the annual Gr by the number of cells produced. **(C)** Correlation between average cell growth rate and tracheid diameter for the example shown in **B** (year 2013 of tree D7, numbers indicate the cell position in the ring); and **(D)** scatter plot of average cell growth rate and tracheid diameter for all 9468 tracheids included in the 250 tracheidograms. The exponential fitting function is shown in red. **(E)** Summary of all correlations between average cell growth rate (Gr) and tracheid diameter (TD) plotted as a function of ring width (*n* = 250). The red dot refers to the example shown in **C**. The red line shows the distribution of the correlations.

An important model assumption is that the main environmental conditions (temperature, light, and soil moisture) occurring when the cells are residing in the cambial zone determine the (future) growth rate (Vaganov et al., 2006). In other words, the growth rate of the cambial zone cells (i.e., the actively dividing cells) cannot be higher then allowed by the most limiting factor. To assign the average cell growth rates necessary to produce the tracheidograms, in this study we make the additional assumptions that (i) the number of dividing cells over a full growing season corresponds to the number of cells produced in the tree ring, and (ii) the actively dividing cells are successively produced with no overlap. Thus, we assigned the growth rate matching each dividing cell by calculating the average growth rates over the time period required to form the corresponding part of the tree-ring (see an example for the year 2013 in tree D7 in **Figure 3B**). Specifically, this time assignment has been performed by splitting the modeled growing season by a number of equally length periods corresponding to the total number of cell produced in the annual ring as quantified with the tracheidogram. This procedure is necessary to identify the period each dividing cell was spending in the cambial zone. Subsequently, the growth rates have been averaged for each period and assigned to the corresponding cambial cell. A corresponding R-code for time-assignment (VS-timing) has been developed for that purpose and applied to derive the linear dependency between the average cell growth rate and the tracheid size (**Figures 3C,D**). This relation is then inversely applied using the fitted function displayed in **Figure 3D** to estimate tracheid size (TD) from the growth rate obtained from the model.

### Modeling Tracheidogram for Each Grouping of Radial Patterns

The previously quantified associations have subsequently been applied to each ring type (N, W, and I) to assess the ability of the model to reproduce the expected pattern of the tracheidogram. Specifically, we run the VS-model with the same original parameterization to calculate the typical seasonal growth rates for each group and subsequently we applied the exponential dependency between growth rate and tracheid size (as described in **Figure 3D**) to obtain the synthetic tracheidograms. As model input we first calculated and then smoothed with a LOESS function (span = 0.3) the daily temperature and precipitation average over the five calendar years included in each ring type. In order to account for the soil moisture legacy, the model was run on a sequence of 3 years, whereby the two first years where feed with the overall daily climate average (1936–2013) and the third year with the group average. Only the output relative to the third year has been considered for further analysis.

### Identifying Threshold Conditions Inducing IADF

To identify the threshold conditions generating IADFs, we compared modeled tracheidograms from newly generated climatic scenarios obtained by progressively increasing the limitation of the main factor inducing the formation of IADF (i.e., by reducing precipitation by step of 20%). The same temperature course (as the average temperature among the groups) has been applied for each scenario to focus on the influence of precipitations only. The occurrence of IADF in the modeled tracheidograms has then been assessed with the same criteria used to its identification (**Figure 2B**).

## Results

### Trees and Tree-Ring Characteristics

The selected *P. sylvestris* trees were on average 17.8 (± 1.4 sd) m tall and their stem DBH varied between 26 and 46 cm. Tree age ranged from 76 to 111 and their average annual radial growth (TRW) over the period 1936–2013 was 1.46 cm (**Table 1**). The common signal, expressed as the mean correlation among the individual detrended time series, was 0.62. The five trees selected for the tracheidograms were highly synchronized with the site chronology (*r* = 0.77) and showed a similar average annual growth of 1.33 cm. In terms of number of cell production, the annual increment was composed on average by 38 radially aligned tracheids per ring (from a minimum of nine to a maximum of 76), but with variation among the trees (mean ranging from 21 to 49 tracheids).

### Model Calibration and Verification

The selected parameters of the calibrated model are shown in **Table 2**. The calibrated model provided a simulated chronology matching the residual tree-ring chronology with a correlation of *r* = 0.71 (*p* < 0.001; *n* = 54 years) and a Gleichläufigkeit Glk of 80% over the calibration period, and a correlation of *r* = 0.55 (*p* < 0.001, *n* = 25, Glk = 71%) for the validation one (**Figure 1**).

**Table 2:** Selected model parameters.

Param	Description	Values
*T_min_*	Minimum temperature threshold for growth (^o^C)	5.0
*T_opt_*_1_	Lower temperatures threshold for optimal growth (^o^C)	13.0
*T_opt_*_2_	Upper temperatures threshold for optimal growth (^o^C)	22.0
*T_max_*	Maximum temperature threshold for growth (^o^C)	32.0
*W_min_*	Minimum soil moisture threshold for growth, relative to saturated soil (v/v)	0.0775
*W_opt_*_1_	Lower soil moistures threshold for optimal growth (v/v)	0.25
*W_opt_*_2_	Upper soil moistures threshold for optimal growth (v/v)	0.375
*W_max_*	Maximum soil moisture threshold for growth (v/v)	0.45
*W*_0_	Initial soil moisture (v/v)	0.15
*T_beg_*	Temperature sum threshold for onset of growth (^o^C)	110.0
*t_beg_*	Size of the moving window for calculation of temperature sum (days)	10
*l*_r_	Depth of root system (mm)	500
*P_max_*	Maximum daily precipitation for saturated soil (mm/day)	40
*C*_1_	Fraction of precipitation reaching the soil (not caught by crown) (rel. unit)	0.5
*C*_2_	First coefficient for calculation of transpiration (mm/day)	0.3075
*C*_3_	Second coefficient for calculation of transpiration (mm/day)	0.11
Λ	Coefficient for water drainage from soil (rel. unit)	0.005
*V_cr_*	Critical growth rate to determine the end of the growing season (rel. unit)	0.04

According to the model, the growing season at the site extended on average for 131 days ± 12, from DOY 137 ± 10 (May 18th) to DOY 268 ± 8 (September 26th). Growth was limited by drought from DOY 139 ± 10 (May 20th) to DOY 259 ± 10 (September 17th), and only affected by temperature limitation at both edges (beginning and end) of the growing season.

### Tracheid Anatomy and Tracheidograms

The average tracheid radial diameter (TD) in the ring differed among trees ranging from 18.7 to 45.5 μm (**Table 1**). Intra-annually, TD usually decreased monotonically from a maximum in the earlywood (∼60 μm) to a minimum in the latewood (∼10 μm, **Figure 2A**), but in some cases it was possible to identify the typical signature of latewood IADF (**Figure 2B**). IADFs occurred mainly in particular years (i.e., 1973, 1995, 2001, 2007, 2009) and mostly when the ring width was >1 mm (**Figure 2C**). The frequency of IADF occurrence was 21.2% (53 rings out of 250) and varied quite strongly among the trees (from 6% in D5 to 46% in D7).

### Calculation of Timing and Growth Rate of Cambial Cells

One assumption of the model is that the conditions occurring at time of formation of cambial cells determine their cell developmental stages. The correlations between the observed cambial and enlarging cells with the modeled growth rate of cambial division is *r* = 0.93 and *r* = 0.84, respectively (**Figures 3A,B**). We used this assumption to first assign a time to each dividing cambial cell to subsequently assign the corresponding average cell growth rate (Gr, as previously described and displayed in **Figure 3B**). As shown in **Figure 3C** and **Supplementary Figure S1**, there is a highly significant correlation (*r* = 0.86, *p* << 0.01 in **Figure 3C**) between average cambial growth rate and tracheid radial diameter. The correlation distribution shows a high frequency of strong significant correlation (*p* < 0.01) (**Figure 3D**). In some rings, however, this relationship clearly weakens. Nonetheless, if in these specific rings the modeled growing season is progressively reduced by excluding the end-of-season growth-rates, the correlations and the R^2^ are considerably recovered (see **Supplementary Figures S2, S3**).

### Modeling and Simulating Growth Rates and Tracheidograms by Ring Type

**FIGURE 4:**
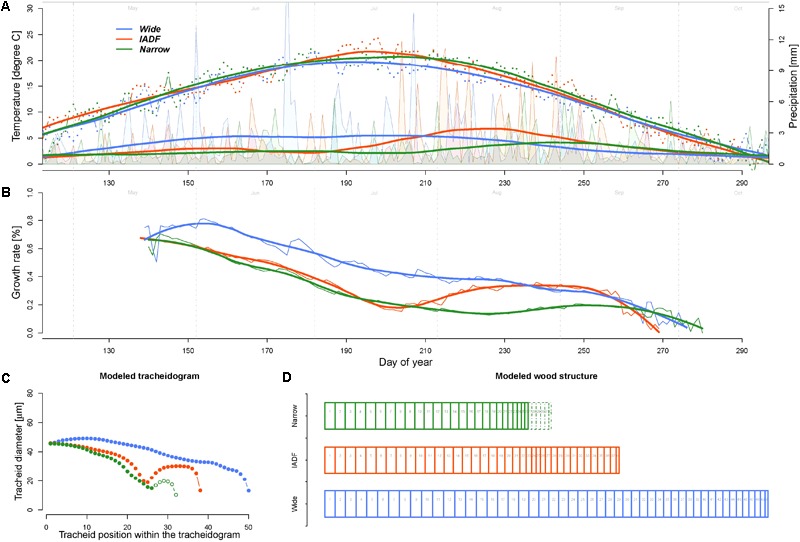
Comparison of climate, growth rate, and modeled wood structure among the three ring types [narrow rings (green); wide rings (blue); IADF rings (red)]. **(A)** Daily temperature (dots) and precipitation (area) averaged for each the year in each ring type and smoothed with a LOESS function (span = 0.3, thick lines). The smoothed values have been used as input for the model. **(B)** Growth rates obtained by the model. **(C)** Derived tracheidogram for each ring type by using the quantified relation between average cell growth rates and tracheid diameter (**Figure 3D**). The number of cell is proportional to the modeled ring width. **(D)** Schema of the tracheidogram shown in **(C)**.

The model calculations of the daily growth rates using the average daily climatic condition of the years of the narrow (N), wide (W), and IADF (I) ring types indicated a common decreasing growth rates between 37% (W) and 64% (N) during the first half of the growing season (up to DOY 210, **Figure 4**). During this period, the rates were about 42 and 48% higher for the wide ring then for the other two groups. The pattern among the groups differed in particular in the second part of the season, where the Grs continued to decrease in the wide rings, while it showed a substantial recovery before a new decrease at the end of the growing season for both IADF (+134%) and the narrow rings (+24%). From DOY 1 to 270 the precipitation sum was 394, 363, and 286 mm for W, I, and N, respectively, whereby the precipitation in the IADF group were initially not substantially differing from the narrow rings, but showed a strong increase in the second part of the growing season. Over the growing season (DOY 142–270), the average temperature among the groups ranged between 16.35°C and 16.64°C.

The assessment of tracheid size (and thus of the average tracheidogram) for each group of year (using the average relationship obtained based on the observation, **Figure 3**) indicated that the model is able to reproduce the main pattern of the original tracheidogram, with presence of IADF only in the corresponding group (**Figure 4C**). However, the model failed to reproduce narrow rings, since growth rate, similarly to the IADF group, also recovered after the summer drought. A stop of growth induced at the end of the drought period would, however, provide a narrow ring, without IADF.

**FIGURE 5:**
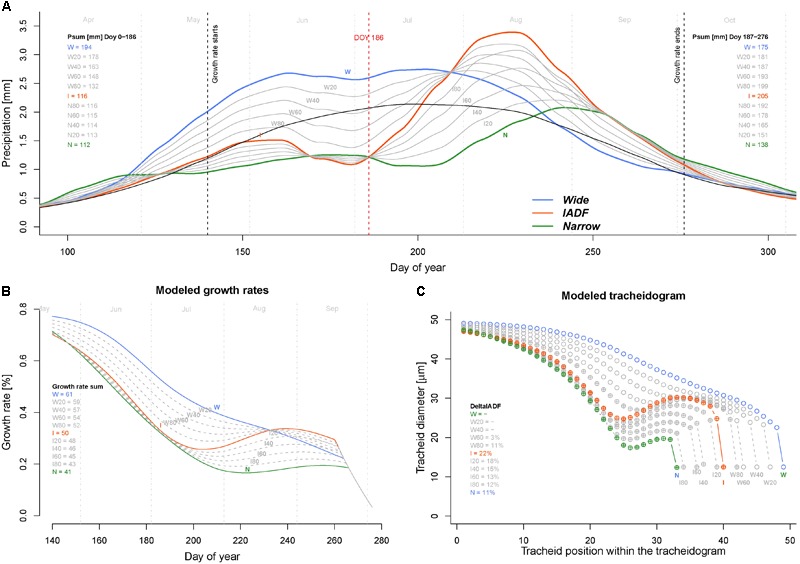
Exploring climatic scenarios generating IADF. **(A)** Averaged and LOESS smoothed (span = 0.3) daily precipitation of each ring type (thick colored lines) and their progressive transition between groups (in 20% steps) between W–I and I–N (thin gray lines) used to run the model (with group average daily temperature). The black line indicates the average precipitation over the period 1960–2013. Psum (mm) Doy 0–186 = precipitation sum between DOY 1 and 186 (middle of the growing season); Psum (mm) Doy187–276 = precipitation sum between DOY 187 and 276 (end of the growing season). **(B)** Obtained daily growth rates. The legend quantifies the growth rates sum (%) for each climatic scenario. **(C)** Derived modeled tracheidograms for each precipitation scenario. Tracheidograms with filled circle display a L+IADF according to the definition given in **Figure 2B**. The legend quantifies the DeltaIADF (%) for each climatic scenario. N = narrow rings (green); W = wide rings (blue); I = IADF rings (red). W20–W80 and I20–I80 indicate the 20% steps of precipitation change between W–I and I–N, respectively.

Model simulations performed to identify which conditions generate IADF by progressively reducing precipitation (by step of 20%) from W to I and I to N during the firs half of the year (period DOY 1–186) indicated that IADF started to appear when precipitations are below 132 mm only if the second part of the growing season get sufficient precipitations (199 mm, see scenario W80 in **Figure 5**). Notably, if the second part of the growing season persists with below average precipitation, the model predicts the formation of a narrow ring.

## Discussion

The application of the VS-model performed in this study, although based on assumptions simplifying the process of xylogenesis, provided outputs matching site observations. It is known that, differently to our assumptions, the xylem cells resulting from cambial zone are disposed in a radial band and thus successively undergo the differentiation program with some time overlap (Rathgeber et al., 2016). Moreover, a latewood tracheid differentiation can easily last for more than a month (e.g., Cuny et al., 2013) and consequently cambial division stops earlier then assumed in this study. Despite these assumptions, the VS-model provided (i) time-series of annual growth rate well matching the site tree-ring chronology, (ii) timing of cambial cells growth rates that matched with the observations of tissue formation performed in the field in 2013 (**Figures 3A,B**), and (iii) modeled tracheidograms reproducing most of the anatomical patterns of tree-rings typical in summer-drought exposed sites.

These results confirm the ability of the VS-model to deliver reliable annual growth outputs at cold and drought limited forest sites (Breitenmoser et al., 2014), as in the Qilian Mountains and Tibetan Plateau (e.g., Zhang et al., 2016; He et al., 2017, 2018; Yang et al., 2017), or within the drought sensitive Mediterranean Basin (Touchan et al., 2012). At our site, growth rates are generally limited by soil water shortage except for the margin of the growing season which are limited by cold (Arzac et al., 2018; Tychkov et al., unpublished), as confirmed by results of climate-growth responses performed for the same species within the South-Central Siberian forest-steppe (Babushkina et al., 2015). Newly, we could verify that the VS-model also provided convincing intra-annual output typical of summer drought-limited environments characterized by a bi-modal growth (e.g., Camarero et al., 2010; Pasho et al., 2012; Vieira et al., 2014) and a radial ring pattern with the occurrence of numerous IADF (e.g., Campelo et al., 2007; De Micco et al., 2016; Zalloni et al., 2016). On the one hand, the growth rates modeled for the 2013 growing season were synchronous in timing and proportion with the number of cambial and enlarging cells observed on micro-cores collected in the field (*r* = 0.93 and 0.84 for cambial and enlarging, respectively), including direct observations on five trees performed in the same area during the years 1979–1981 (Vaganov et al., 1985). On the other hand, the tracheidograms simulated for the three ring types provided the expected radial patterns, with the occurrence of IADF in the second part of the annual ring for the IADF group.

However, interestingly, the model failed to reproduce the tracheid pattern of narrow rings by instead providing narrow rings with the occurrence of IADF. Strong indications suggest that this discrepancy is caused by a summer drought-induced growth stop in that particular year that the model misses to identify. In this specific case, there are indications that in extreme drought years, trees with reduced growth rate are not able to resume growth along with the drought release occurring in the second part of the season due to an excessive water shortage. This result is confirmed by previous observations indicating that water availability has a strong influence on growth rates and can induce an early cessation of wood formation (Eilmann et al., 2009; Vieira et al., 2017). Studies on IADF occurrence have already highlighted that IADFs were more frequent in younger trees (Vieira et al., 2009; Battipaglia et al., 2010) or in wider tree rings (Rigling et al., 2001; Campelo et al., 2013), supporting our suggestion that trees with smaller growth rates do not resume cambial division and thus do not form IADFs. The substantial improvement of the correlations and R^2^ between cambial cell growth rates and cell diameter obtained when reducing the modeled growing season length (**Supplementary Figures S2, S3**) well supports our hypothesis.

The results obtained also reveals that the VS-model can be used for describing the processes underlying the environmental impact on the intra-annual tree-ring structure, at least within contexts characterized by climatic factors strong limiting growth, as at our site. In particular, the strength of the association between the simulated growth rates and the measured radial tracheid sizes supports the model assumption that tracheid radial size is pre-determined during the early stage of cell differentiation and can therefore be associated with the rate of cambial cells production (Vaganov et al., 2006, 2011). The climatic conditions occurring during this phase pre-determine the duration and rate that the forming cells are going to endure in the enlargement phase, which control the final tracheid size (Cuny et al., 2014). Studies on correlation between tracheid size and environmental conditions have indeed often identified seasonal climatic signals overlaying the developmental phases of cambial division and tracheid enlargement, thus supporting the existence of a strong association between them (e.g., Fonti et al., 2013; Carrer et al., 2017; Castagneri et al., 2017).

In this study we applied the model to assess which conditions (intensity and pattern of precipitation) generate a latewood IADF in “an average tree” at the study site. Such an approach has a great potential for exploring how climate is affecting wood formation, even at individual tree level. For example it might be interesting to investigate what summer drought conditions are inducing a stop in growth while considering both the level of drought and the individual tree growth potential by performing similar analyses on trees and calendar years grouped according to ring width and/or tree vigor.

## Conclusion

This study demonstrated that the VS-model successfully generated realistic tracheidograms of tracheid cell diameter for *P. sylvestris* trees growing in a drought sensitive environment. In particular the strong association between the growth rate of the dividing cambial cell and the tracheid size was used to predict wood structure from daily climatic condition This intra-ring resolution has been achieved thanks to the model ability to provide daily growth rates (Vaganov et al., 2006). This increased resolution helped us to identify narrow rings as an extreme manifestation of IADFs where the recover in the second part of the season was impeded by a too high sensitivity to extreme drought conditions. The model proved usefulness also to quantify average levels and seasonal patterns of precipitation (thus indirectly of drought) inducing IADFs and narrow rings. The association between climatic conditions and cell anatomical structure via model-generated tracheidogram provide a novel opportunity to assess wood structure sensitivity to climate. Such intra-annual growth model resolution represents a fundamental tool to provide reliable tracheidogram to better understand, develop and up-scale scenarios of wood structural responses to climate change over time and space. Considering that wood structure is an important legacy for tree performance (Björklund et al., 2017; Rathgeber, 2017) the results from those scenarios might have relevant implications for the assessment of future plant productivity and provided forest services (Sass-Klaassen et al., 2016).

## Author Contributions

MP, EV, VS, and PF designed the research. EB and MF performed data collection. MP, MF, and PF performed analyses and interpreted the data. All co-authors contributed to the preparation of the manuscript.

## Conflict of Interest Statement

The authors declare that the research was conducted in the absence of any commercial or financial relationships that could be construed as a potential conflict of interest.

## Supplementary Material

FIGURE S1 Relationship between average cell growth rate and tracheid diameter. Left panels: Tracheidograms (black solid line) and smoothed integral growth rate (gray solid line) obtained for five individual trees for the annual ring 1987. Right panels: Scatterplots between daily growth rates and the radial cell sizes and obtained linear regressions with estimated R^2^.

FIGURE S2 Example of how the reduction of the growing season length improves R^2^ between the average cell growth rate (Gr) and tracheid radial diameter (TD). Data shown refers to the tracheidogram of the tree-ring 1974 in Tree 5. Left graphs: Radial tracheid diameters are indicated with black dots and smoothed with a black line; gray line represents the modeled daily growth rates. Rights graph shows the relationships between cell growth rate and tracheid radial diameter. The R^2^ of the linear regression is indicated. From top to bottom the length of the growing season is progressively reduced by steps of 20-days.

FIGURE S3 Example of how the reduction of the growing season length improves the R^2^ between cell growth rate and tracheid radial diameter for all 250 tracheidograms. **(A)** Gray area indicates linear regression (R^2^ < 0.4) for the years with narrow tree rings. **(B)** R^2^-values as a function of duration of the growing season. **(C)** Transformed R-distribution after reduction of growing season.
