# Supplementary figures and images for: Modeled Tracheidograms Disclose Drought Influence on Pinus sylvestris Tree-Rings Structure From Siberian Forest-Steppe
Source: Front Plant Sci. 2018 Aug 6;9:1144. doi: 10.3389/fpls.2018.01144 (PMC6088211; doi:10.3389/fpls.2018.01144)

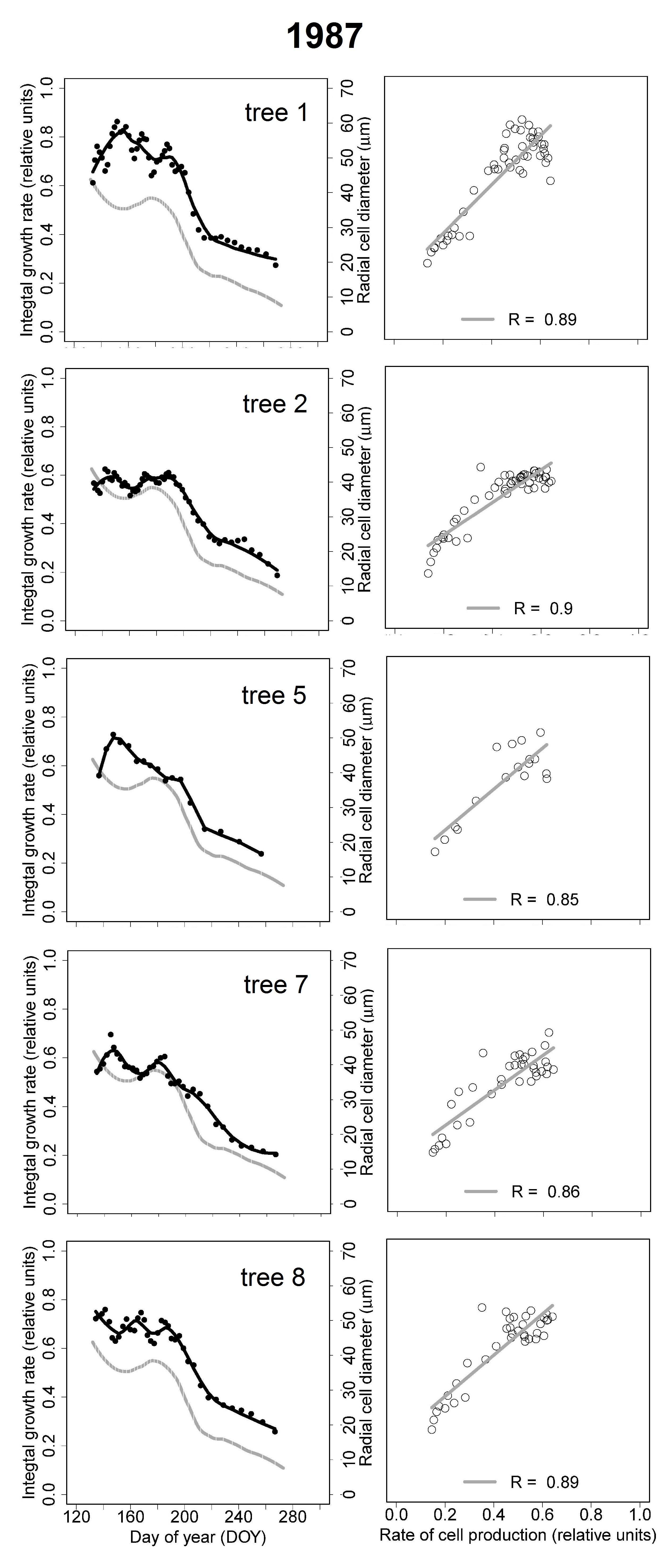

Supplement: FIGURE S1 — Relationship between average cell growth rate and tracheid diameter. Left panels: Tracheidograms (black solid line) and smoothed integral growth rate (gray solid line) obtained for five individual trees for the annual ring 1987. Right panels: Scatterplots between daily growth rates and the radial cell sizes and obtained linear regressions with estimated R2. [file Image_1.JPEG]

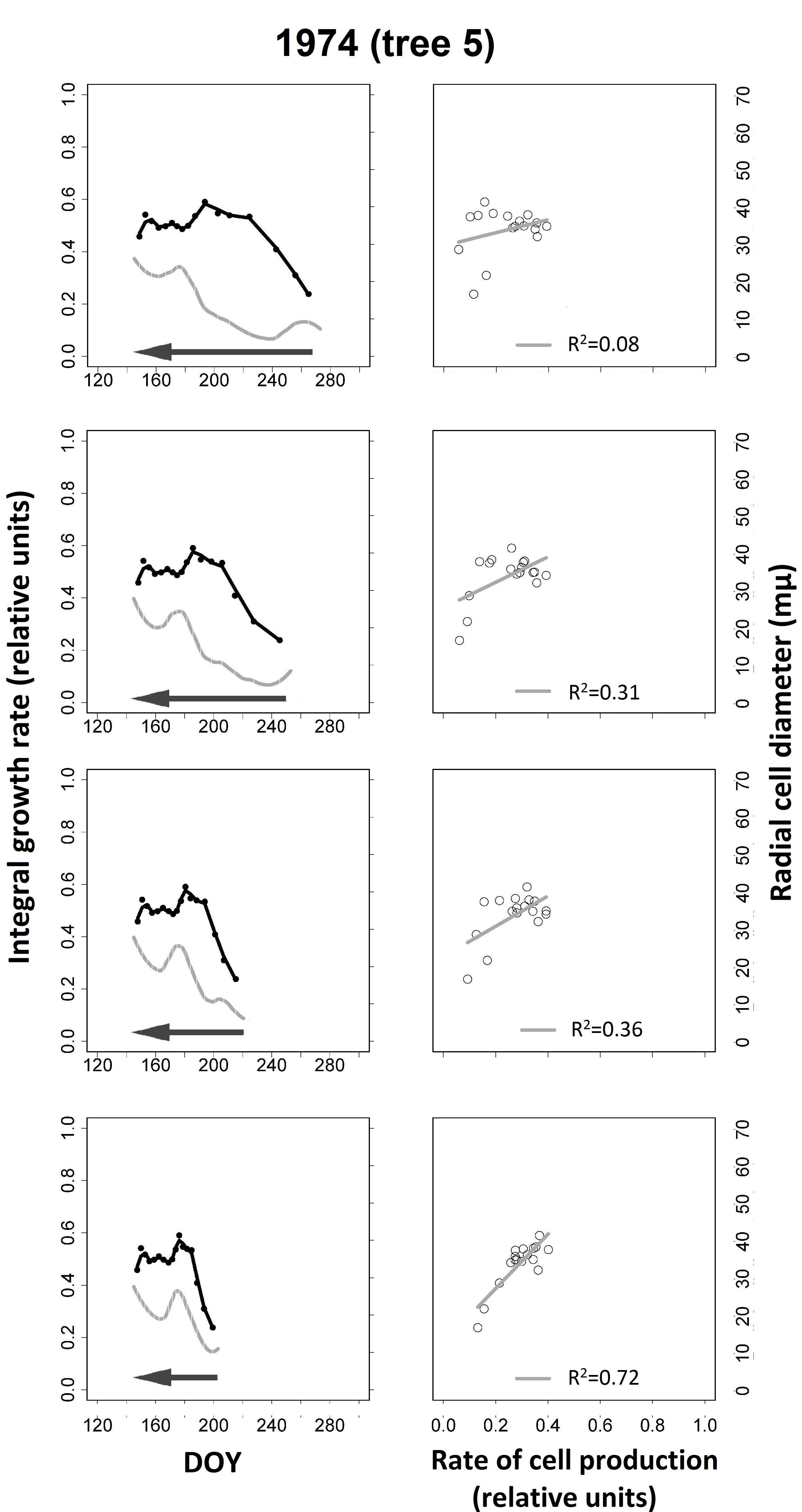

Supplement: FIGURE S2 — Example of how the reduction of the growing season length improves R2 between the average cell growth rate (Gr) and tracheid radial diameter (TD). Data shown refers to the tracheidogram of the tree-ring 1974 in Tree 5. Left graphs: Radial tracheid diameters are indicated with black dots and smoothed with a black line; gray line represents the modeled daily growth rates. Rights graph shows the relationships between cell growth rate and tracheid radial diameter. The R2 of the linear regression is indicated. From top to bottom the length of the growing season is progressively reduced by steps of 20-days. [file Image_2.JPEG]

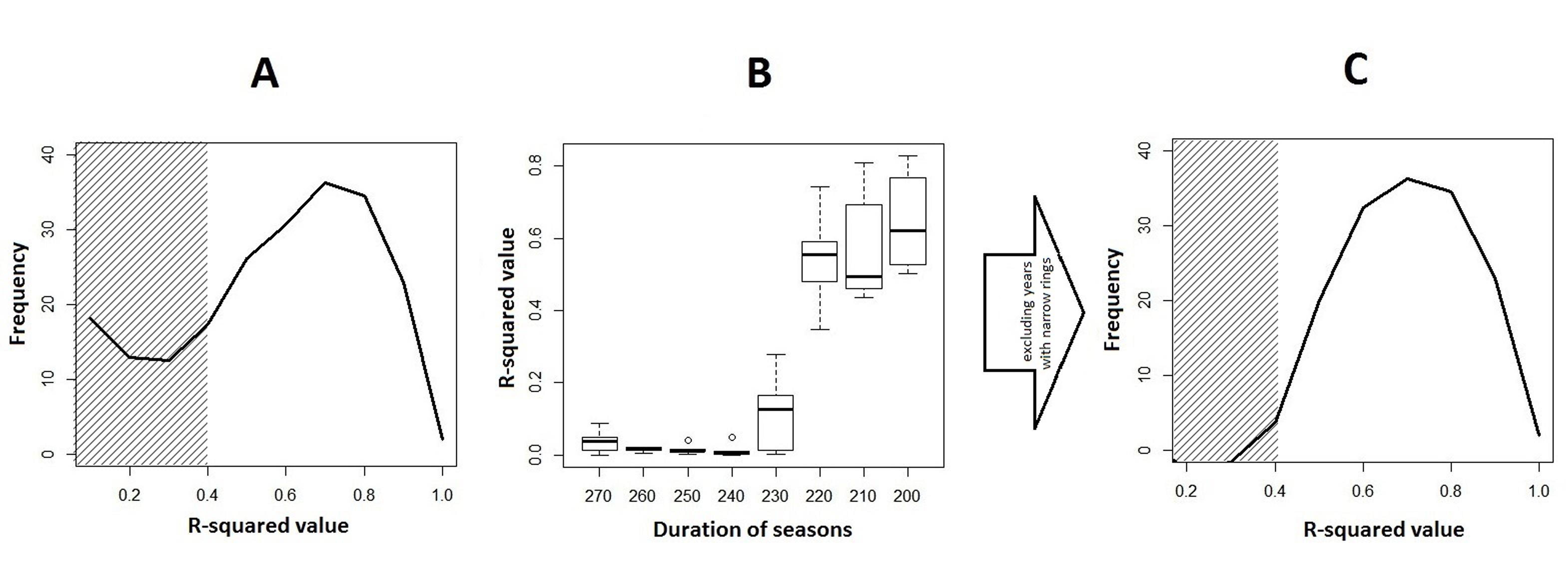

Supplement: FIGURE S3 — Example of how the reduction of the growing season length improves the R2 between cell growth rate and tracheid radial diameter for all 250 tracheidograms. (A) Gray area indicates linear regression (R2 < 0.4) for the years with narrow tree rings. (B) R2-values as a function of duration of the growing season. (C) Transformed R-distribution after reduction of growing season. [file Image_3.JPEG]
